# Reports on the Hospitals of the United Kingdom

**Published:** 1910-02-05

**Authors:** Henry Burdett


### REPORTS
ON THE
### Hospitals of the United Kingdom.

By SIR HENRY BURDETT, K.C.B., K.C.V.O.

### XXIII.—MORE WELSH HOSPITALS.

### SWANSEA GENERAL AND EYE HOSPITAL.

This hospital presents an attractive appearance when seen from the wards. It stands on a self-contained site, which will enable the managers to provide adequate and complete verandahs, large enough to take the beds from the wards, and to leave ample space on the verandahs to permit the staff to discharge their duties in comfort. A beginning has been made on the ground floor by providing a platform with an awning; but this is not wide enough or large enough for the number of patients in each ward. The surgical wards are overcrowded by the addition of a number of beds in excess of the accommodation to which each ward should be confined in a well-administered hospital. This excess is increased at night by the admission of new cases, mostly accident, the effects of which system could be readily modified at relatively little cost if each of the wards had attached to it a large verandah facing west or south, covered by a glass roof, so that the patients could sleep out of the wards on the verandah. Their recovery should thus be expedited and the atmospheric condition of the wards be improved to a material extent. We spoke to patients on the verandah, such as it is at present, who expressed their delight at the improved condition of their health and the comfort they had experienced since they had lived for many hours in the open air. There are many prosperous citizens in Swansea, and if one of them would set the example by providing one ward, with an up-to-date and spacious verandah running the whole length of the main surgical ward, others would soon follow his example, we have little doubt. At any rate verandahs should be forthcoming of the type we have described without further delay. Unfortunately the expenditure of the hospital exceeds £9,500 a year, and the invested property is under £40,000. The Committee has therefore no spare funds in hand for building and improvements—an excellent reason why a shrewd man of business should look into this matter, take it up energetically and not rest until the verandahs are provided throughout the hospital.

Another urgently required building is a new, well-planned, up-to-date casualty and out-patients' department. The present provision is most inadequate, and if not speedily changed must become a grave scandal. Again, the present nurses' home is very badly planned, and is indeed about the worst building of the kind we have inspected in the South and West of England. It is most inconvenient to work, and has few features of comfort or attraction. More accommodation is urgently called for, and we counsel the Board to re-establish a private staff of nurses, and to set apart the existing home for the use of this staff alone. This will enable the board to have plans prepared embodying a well-thought-out scheme, whereby the new nurses' home for the hospital staff proper can be made so complete as to constitute it a type of what a nurses' home ought to be in the present day. Nurses should have the best accommodation, seeing that their work in a hospital is arduous and exhausting; and the crowded state of the surgical wards at Swansea makes the Committee's responsibility in this matter peculiarly heavy.

The Committee has shown its sense of the importance of making the hospital buildings as efficient as possible by causing new and up-to-date fittings to be introduced into the lavatories, and by the reconstruction and improvement of most of the larger wards. These improvements have repaid the cost entailed many times already by the improved hygienic conditions in these wards, so making them infinitely more efficient for the treatment of disease.

The condition of the verandahs, so-called, which have recently been introduced is not satisfactory for so large a hospital, for they are neither wide enough nor nearly long enough really to relieve the crowded wards, facts which the medical staff should bring home to the General Committee, so as to secure that adequate accommodation may be forthcoming in the near future.

It was a pleasure to notice the active and zealous way in which some of the sisters discharge their duties. Notably the sister in charge of the main surgical ward and operation theatre. It is not easy to maintain a cheerful countenance when overworked; a fact the recognition of which by hospital managers would often do real service to the general efficiency of the hospital under their control.

This hospital has a wing devoted to ophthalmic cases, of which there appears to be an unusually large number. The department is well organised and administered, and must be a great boon to the working classes throughout the district served by this hospital. The patients throughout the hospital were markedly happy and comfortable, facts which testify to the kindness they receive at the hands of the medical and nursing staffs, and indeed from all with whom they have to do whilst resident in the Swansea General Hospital.

#### The Private Nursing Staff.

There was formerly a staff of private nurses at the Swansea General Hospital. In recent years the Committee has permitted this safeguard for its subscribers and the public of the district to be given up. This was certainly a serious error on financial and public grounds, for in these days when the action of Parliament is having the effect of diminishing that sympathy between class and class, which has done so much for Great Britain in the past, every hospital committee must be actively alert to counteract the effects of this action by entering into closer relations with its governors and supporters. This can best be done by maintaining an efficient private staff of nurses, and giving the first call upon their services to subscribers and governors. That should mean a large addition to the subscription list; and so the financial position of the hospital would be strengthened immensely. Economical considerations are calculated to enforce a standard cost per bed and per patient every year in the near future. One effect of this will be that the number of probationers each hospital trains must then be limited to the actual requirements of each hospital. So a good private staff of nurses will become essential to every voluntary hospital, for in no other way can the economic conditions be fulfilled. Such a private staff, if the governors had the first call upon the services of the nurses, would soon increase the number of annual subscribers materially.

### ABERGAVENNY (THE VICTORIA) COTTAGE HOSPITAL.

The present Hospital occupies a new building built so recently as 1902, but contains no bathrooms for the staff or the patients. In one small room there are two portable baths but no hot-water supply, or waste. This important defect astonished us, as the Hospital in other respects, save the mortuary, is well found and well kept.

The Hospital contains seven beds and a cot in two wards with three beds each, one for men and one for women, and one single-bedded ward. All of these are bright and airy. The mortuary, so-called, has no window, no cemented walls and no facilities for post-mortems. At present it shows neither reverence for the dead nor any regard for the living, as represented by the medical staff and the requirements of pathology.

This Hospital has an attractive appearance: it is surrounded by ample grounds and a pleasant garden, and its popularity, despite the defects which we have pointed out, may be judged by the fact that all the beds are constantly occupied. The nursing staff consists of two trained nurses and a matron, so that the patients ought to be over rather than under nursed. Hanging in the hall is a map of England and Wales, with every cottage hospital marked upon it. It is the work of Mr. D. Howell James, the Treasurer, and is full of interest. We congratulate Mr. Howell James upon its production, for it must have caused him considerable trouble to complete.

### MERTHYR GENERAL HOSPITAL.

This is a hospital which ministers to the needs of the miners of a large district. It has no special features, and was not built, we imagine, from the plans of an architect familiar with hospital requirements. Parts of it might be reconstructed with advantage, and it is an anachronism that the matron who has the whole responsibility of its administration, there being no resident medical officer, should not have a comfortable and spacious room for her own use. She has only a small slip of an apartment opening into the dispensary. This in our judgment is improper; as the principal resident officer she ought to have suitable apartments, and the present room, if room it can be called, might serve as her office, especially as she has the responsibility of looking after a private nursing staff.

The Hospital consists of two large wards, one of which is divided in the middle by a glass partition, one half being set apart for the children, the other for the women. Some of the baths are very unsightly, and require relacquering or renewal. There is an accident ward of five beds with very elaborate encaustic tiles, and a large annexe also with encaustic tiles which contains a really horrible bath and very little else. Anything more purposeless and useless than this annexe we have never seen in a hospital.

A nurses' home, which has just been completed, had not yet been furnished, and had to be made ready for use. There are 16 nurses, of whom four are on the private staff. The impression the Hospital gave us was that it is too sleepily conducted, and wants smartening up.

### XXIV.—SOME COTTAGE HOSPITALS.

### MALMESBURY COTTAGE HOSPITAL.

This building, a comparatively new one (1897), illustrates most points which an architect should avoid in attempting to plan a hospital. The site lent itself to a good, or rather to an adequate plan, for there are only nine beds and two cots. Unfortunately, everything but the elevation seems to have been deemed of little importance, and many an old building adapted for hospital purposes is less faulty and objectionable than this relatively new one. The baths and w.c.'s are just what they should not be hygienically and otherwise, and are, in fact, the worst possible. We are sorry to express our conviction that this is probably the worst planned cottage hospital in the country. We do not know who is responsible for this, for the elevation is most attractive and well in accord with its surroundings. The staff appear to have done what they can to make the best of the building as they find it. Here, too, the mortuary is bad. It is used as a store-house for couches, etc., and we found a body there too when we inspected it on September 2, 1909. Such things should not be possible in a hospital, where reverence for the dead should always be shown by the living.

### WELLINGTON AND DISTRICT COTTAGE HOSPITAL, SOMERSET.

This hospital was established in 1892, through the generosity of Mr. Egerton Burnett, whose factory adjoins the hospital grounds. It contains eight beds and a cot, but having two stories and covering much ground it gives the appearance of a much larger hospital. There are two wards, that for men being downstairs, and that for women on the first floor. It was not designed by a hospital architect, or it would have been less costly, more compact, and easier to work. At first it contained no bathroom, and the operation theatre, which is well fitted but badly lighted, is also the dispensary, so that the plan for hospital purposes presents few or no merits. Still the accommodation is ample, and everything is spotlessly clean and well-kept. The matron, whose name is not mentioned in the report—a strange omission—was at Crumpsall for seven years. She knows her work, and keeps everything in good order. The patients were evidently well looked after and comfortable. Generally it may be said that no money has been spared on buildings, and that it is matter for regret that expert knowledge was not sought and used before the plans were agreed to.

The **Royal Halifax Infirmary** and the **Bolton Infirmary and Dispensary** will be reported on next week.

